# Supplementary material for: Content-rich biological network constructed by mining PubMed abstracts
Source: BMC Bioinformatics. 2004 Oct 8;5:147. doi: 10.1186/1471-2105-5-147 (PMC528731; doi:10.1186/1471-2105-5-147)
Supplement: Additional File 2 — The original results of the above study (non-essential files are deleted to keep the file size under the limit set by BMC bioinformatics). [file 1471-2105-5-147-S2.bz2 › chilibotAdditionalFile2/dip20/725ID9822594E18074/html/HLYD_HLYB.html]

 


 **HLYD** and **HLYB** 
  
Found 77 abstracts in PubMed, retrieved 20.  
 

 What does Google say? 
 PDF only 
| .edu only 

---

**Interactive relationship** (e.g. stimulation, inhibition, etc)

**Neutral relationship**- This suggests that the presence of  **HlyB**  induces a structural change in  **HlyD** .  Ref: 10071218 Mol Gen Genet, 1999
- These results are consistent with an interaction between  **HlyD**  TolC and  **HlyB**   **HlyD** .  Ref: 10071218 Mol Gen Genet, 1999
- and b the C terminal sequence of  **HlyD**  interacts with periplasmic loop s of  **HlyB**  to form a closed channel spanning the periplasm.  Ref: 9393456 Mol Gen Genet, 1997

**Non-interactive relationship** (e.g. studied together, co-existance, homology, etc.)

- This PagC HlyAs fusion protein was expressed and efficiently secreted into the culture supernatant by E. coli upon complementation with the hemolysin secretion proteins  **HlyB**  and  **HlyD** .  Ref: 8766698 Appl Microbiol Biotechnol, 1996
- Moreover, they suggest that the  **HlyB**   **HlyD**  TolC based antigen delivery system with attenuated Salmonella spp.  Ref: 9119463 Infect Immun, 1997
- **HlyB**  and  **HlyD**  are involved in secretion of the toxin.  Ref: 9521785 Biochemistry, 1998
- **HlyD**  and  **HlyB**  assembled a stable IM complex in the absence of TolC and substrate.  Ref: 9822594 EMBO J, 1998
- Our data suggest that all beta Gal HlyAs fusion proteins generated are secreted, albeit with varying efficiencies, by the  **HlyB**   **HlyD**  TolC hemolysin secretion machinery under Sec proficient conditions.  Ref: 8842146 Mol Gen Genet, 1996
- The system which consists of the two specific membrane proteins  **HlyB**  and  **HlyD**  and the outer membrane protein TolC, recognizes on HlyA a C terminally located signal sequence of about 60 amino acids.  Ref: 9382730 Behring Inst Mitt, 1997
- Our data suggest that TnhlyAs generated hemolysin fusion proteins are secreted efficiently by the  **HlyB**   **HlyD**  TolC hemolysin secretion machinery.  Ref: 9711856 FEMS Microbiol Lett, 1998
- A derivative of  **HlyD** , HlyD22, lacking the 40 N terminal residues of  **HlyD**  assembles into the inner membrane displaying the same stability with and without  **HlyB**  as wild type  **HlyD**  does.  Ref: 10071218 Mol Gen Genet, 1999
- We show that an scFv HlyA hybrid secreted by Hly type I transporter TolC,  **HlyB** ,  **HlyD**  is accumulated in the extracellular medium with the disulphide bonds correctly formed.  Ref: 11309117 Mol Microbiol, 2001
- A recombinant r Salmonella typhimurium aroA vaccine that secretes the naturally secreted protein of Mycobacterium bovis strain BCG, Ag85B, by means of the  **HlyB**   **HlyD**  TolC export machinery termed p30 in the following was constructed.  Ref: 10727883 FEMS Immunol Med Microbiol, 2000
- The export of Escherichia coli hemolysin across the cytoplasmic and the outer membranes requires the COOH terminal signal sequence of HlyA, the two specific translocator proteins  **HlyB**  and  **HlyD** , and the outer membrane protein TolC.  Ref: 9630987 Nat Biotechnol, 1996
- In addition,  **HlyB**  itself appears to be less stable in the absence of  **HlyD** .  Ref: 10071218 Mol Gen Genet, 1999
- In the absence of  **HlyB** ,  **HlyD**  is also unstable and specific degradation products are detected, which co fractionate with the inner membrane, indicating in this case limited cleavage at specific sites.  Ref: 10071218 Mol Gen Genet, 1999
- We engineered plasmid pETR14, containing the  **hlyB**  and  **hlyD**  genes of the Escherichia coli hemolysin operon, to express a fusion protein containing 720 amino acid residues from the nontoxic, receptor binding, carboxy terminus of C. difficile toxin A and the secretion signal of E. coli hemolysin A.  Ref: 9199470 Infect Immun, 1997
- On the contrary, in the absence of both  **HlyB**  and TolC the half life of  **HlyD**  is approximately 110 min.  Ref: 10071218 Mol Gen Genet, 1999
- Second site suppressor mutations in  **hlyD**  and  **hlyB**  were obtained, which abolish the hypersensitive phenotype caused by the 3 terminal mutations in  **hlyD** .  Ref: 9393456 Mol Gen Genet, 1997
- The structure and function of the components of the HlyA secretion apparatus,  **HlyB** ,  **HlyD**  and TolC, have been studied in great detail.  Ref: 11755084 Trends Microbiol, 2002
- To this end, Salmonella typhimurium SL7207 was used as carrier for secreted p60 HlyA fusion protein by Escherichia coli  **HlyB**  and  **HlyD**  transport proteins.  Ref: 7729919 Infect Immun, 1995
- This complex comprises a traffic ATPase  **HlyB**  and the 478 residue adaptor protein  **HlyD** , which contacts TolC during recruitment.  Ref: 11676535 J Mol Biol, 2001
- The genetic determinants for a fusion of its B subunit StxB, which mediates toxin binding to target cells, with the COOH terminus of Escherichia coli hemolysin A Stx HlyA has been combined with the determinants of the accessory translocator proteins  **HlyB**  and  **HlyD**  in a Notl cassette.  Ref: 8905616 Microb Pathog, 1996
- We have developed an export cloning system that is composed of two vectors one in which the fusion of the desired gene with the 3 end of hlyA is generated, and a second in which the sequences containing the fusion are combined with the accessory genes  **hlyB**  and  **hlyD** , thereby reconstructing the natural organization of the hly locus.  Ref: 9630987 Nat Biotechnol, 1996
- A recombinant r Salmonella typhimurium aroA vaccine strain was constructed which secretes the naturally somatic protein of Listeria monocytogenes, superoxide dismutase SOD, by the  **HlyB**   **HlyD**  TolC export machinery.  Ref: 9119463 Infect Immun, 1997
- The resulting 53 kDa hybrid protein was specifically secreted to the external medium in the presence of the haemolysin translocator proteins,  **HlyB**  and  **HlyD** .  Ref: 7500946 Mol Gen Genet, 1995
- The toxin HlyA is exported from Escherichia coli, without a periplasmic intermediate, by a type I system comprising an energized inner membrane IM translocase of two proteins,  **HlyD**  and the traffic ATPase  **HlyB** , and the outer membrane OM porin like TolC.  Ref: 9822594 EMBO J, 1998
- these were termed EHEC  **hlyB**  and EHEC  **hlyD** .  Ref: 8936317 Microbiology, 1996
- This result shows that in the presence of  **HlyB**  removal of TolC renders  **HlyD**  more unstable than it is in the absence of both  **HlyB**  and TolC.  Ref: 10071218 Mol Gen Genet, 1999
- These data suggest that the  **HlyB**   **HlyD**  TolC based antigen delivery system with attenuated r S. typhimurium as carrier is capable of inducing an immune response against mycobacterial antigens.  Ref: 10727883 FEMS Immunol Med Microbiol, 2000
- In addition, we report on our r Salmonella typhimurium expression system combined with the  **HlyB**   **HlyD**  TolC export machinery for delivering the prominent mycobacterial antigen Ag85B for immune recognition.  Ref: 10076914 FEMS Immunol Med Microbiol, 1999
